# Teledermatology in an emergency department: benefits and gaps

**DOI:** 10.1186/s12873-023-00854-2

**Published:** 2023-10-04

**Authors:** Adinia Santosa, Zisheng Li, Nisha Suyien Chandran

**Affiliations:** 1 Division of Dermatology, Department of Medicine, National University Hospital, 5 Lower Kent Ridge Road, Singapore, 119074 Singapore; 2 Emergency Medicine Department, National University Hospital, Singapore, Singapore; 3 Department of Surgery, Yong Loo Lin School of Medicine, National University of Singapore, Singapore, Singapore; 4 Department of Medicine, Yong Loo Lin School of Medicine, National University of Singapore, Singapore, Singapore

**Keywords:** Telemedicine, Health resources, Emergency Medicine, Dermatology

## Abstract

**Background:**

Teledermatology has provided new avenues through which dermatologists can provide healthcare. Teledermatology was introduced to the Emergency Department (ED) to enable immediate dermatological consult. We aimed to assess the impact of teledermatology on the management of dermatological conditions by emergency medicine physicians and subsequent health resource utilization.

**Methods:**

We conducted a retrospective review of teledermatology referrals from the ED of our tertiary hospital in Singapore from June 2015 to December 2019. The dermatological conditions, the triaging and treatment recommendations were analyzed. Follow-up plans were recorded.

**Results:**

Between June 2015 and December 2019, 147 patients were referred from the ED via teledermatology; 11 (7.5%) were admitted, and 136 (92.5%) were recommended to be discharged with a dermatological diagnosis and management plan. If required, a follow-up appointment in the dermatology specialist clinic was arranged. Of the 136 patients who were discharged, 129 (94.9%) patients were provided with outpatient appointment in the dermatology clinic, out of which 110 patients returned for follow-up. 90 (81.8%) patients retained the initial teledermatology diagnoses and 20 (18.2%) patients had their teledermatology diagnoses revised after in-person review.

**Conclusions:**

Teledermatology allows for more efficient triaging of patients with dermatological conditions. Reliability between teledermatology and clinic-based examination is good. Patients may be managed mainly in the outpatient setting with appropriate specialty-directed treatment, return advice, and appropriately-triaged follow-up outpatient appointment.

## Background

Telehealth is the provision of remote health-related services where information and communication technologies are used to exchange information between health provider and patient. Teledermatology, a subset of telehealth, enables review and management of patients with specialist dermatological knowledge without a traditional face-to-face consultation. This allows easier and more convenient access to dermatology specialist opinion with better utilisation of dermatology resources. There are three different modalities of teledermatology care delivery platforms: asynchronous, synchronous and hybrid [1]. The asynchronous method requires taking photographic images or video files of patients and sending them to a dermatologist who responds at a later time, while the synchronous method refers to real-time video teleconferencing. Hybrid teledermatology includes platforms that combine synchronous and asynchronous teledermatology modalities [1].

The majority of specialist dermatology services are delivered through the outpatient model. Patients seeking advice for dermatological conditions are largely reviewed in the clinic setting [2–4]. However, instead of the dermatology outpatient clinic, the emergency department (ED) of a hospital is where patients with dermatological conditions, ranging from mild to severe, may first present [5]. A survey of ED physicians in the United States of America demonstrated that the majority considered a teledermatology service useful in the diagnosis and management of skin diseases and enhancement of patient care [6]. Facilitating the availability of an immediate dermatology specialist input would reassure ED physicians and provide educational benefit, result in timely and appropriate dermatological care, and avoid the admission of patients solely for specialist consultation and management.

Between 2015 and 2019, the ED of our tertiary public hospital reviewed and admitted an annual average of 110,428 patients and 36,650 patients, respectively. The average bed occupancy rate in the wards during this period was 85.9%.

As part of a hospital-wide quality improvement project intended to reduce admissions for dermatological conditions and to allow more optimization of triaging of patients with dermatological conditions, we instituted an asynchronous teledermatology service initiative with the ED.

We aimed to assess the impact of teledermatology on management of dermatological conditions by ED physicians and subsequent health resource utilization.

## Methods

The teledermatology service was offered to ED physicians on weekdays between 9 AM and 5 PM. Any patient with a dermatological condition who was agreeable for teledermatology consultation was offered the service. In the ED, pictures of the skin condition were taken on a dedicated camera and uploaded to the encrypted hospital-wide software together with the relevant clinical history of the patient. The teledermatology referral was initiated via a call to the Dermatology division, and the dermatology team then reviewed the information and responded within 2 h. Further information was requested from the ED physicians as necessary. If the diagnosis was clear, a management plan with return advice and an appropriate outpatient follow-up appointment was given. If the diagnosis was equivocal, the patient would be seen in the Dermatology Clinic in person, on the same day.

All teledermatology referrals from the ED to the Dermatology unit of the National University Hospital between June 2015 and December 2019 were analyzed and anonymized. The number and types of patient admissions to the inpatient wards were collated. Similar parameters were collated for patients discharged directly from the ED. For the latter group, follow-up outpatient appointments and duration to these were recorded. Diagnosis and treatment recommendations were analyzed. Institutional review board approval was obtained. Data collated was checked to ensure credibility.

## Results

There were 147 teledermatology referrals made by the ED over the studied period. 76 (51.7%) of the referred patients were male and 71 (48.3%) female with an age range of 17 to 81 years.

The average length-of-stay (LOS) in the ED for patients that were discharged after teledermatology review is 3 hours and 46 minutes. Disregarding the point during patient’s ED stay at which the teledermatology referral was made, the teledermatology team was able to start reviewing the patient on average within 2 hours and 13 minutes from the start of the patient’s ED stay. Of the 147 referrals, 136 patients (92.5%) could be discharged directly from the ED and managed in an outpatient setting, while 11 patients (7.5%) required inpatient admission. Details of the number of follow-up outpatient follow-up appointments and interval of appointments given are listed in Table 1.

**Table 1:** Recommended disposition of patients referred via teledermatology

Recommendation for 147 asynchronous teledermatology referrals	Patient count (n (%))
Outpatient management †	136 out of 147 (92.5%) Of the 136 patients for outpatient management: > 81 (59.6%) given outpatient follow-up appointment within a week > 39 (28.7%) given outpatient follow-up appointments between a week to two months > 9 (6.6%) given outpatient follow-up appointment but instead admitted for non-dermatological reasons > 4 (2.9%) did not require any further outpatient follow-up appointments > 3 (2.2%) on follow-up with other institutions and did not require further review in our institution
Inpatient management	11 out of 147 (7.5%) Of the 11 patients for inpatient management: > 10 (90.9%) admitted under Dermatology service > 1 (9.1%) suggested for admission but patient discharged himself at own risk

† No patients required admission from clinic during the outpatient follow-up appointment

Analyzing the nature of dermatological conditions referred from the ED via teledermatology and the eventual diagnoses would have an impact on the feasibility and subsequent widespread usage of teledermatology. The list of diagnoses made after teledermatology consult is shown in Table 2. The most prevalent conditions diagnosed were eczema (36.1%) and urticaria (10.9%).

**Table 2:** Dermatological diagnoses of ED patients referred through teledermatology

Diagnosis (n = 147)	Frequency of diagnosis (n (%))
Eczema	53 (36.1%)
Urticaria	16 (10.9%)
Infections of the skin and subcutaneous tissue (impetigo, cellulitis, lymphadenitis, viral, fungal and bacterial cause)	16 (10.9%)
Papulosquamous disorders (psoriasis, lichen planus, pityriasis rosea)	11 (7.5%)
Blistering disorders (pemphigus, pemphigoid)	11 (7.5%)
Vasculitis	9 (6.1%)
Exanthem	5 (3.4%)
Insect bite reaction	4 (2.7%)
Scabies	4 (2.7%)
Erythema Multiforme	3 (2.0%)
Tumours (seborrhoeic keratosis, basal cell carcinoma, squamous cell carcinoma)	3 (2.0%)
*Miscellaneous*	3 (2.0%)
Pruritic urticarial papules and plaques of pregnancy	2 (1.4%)
Cutaneous adverse drug reaction	2 (1.4%)
Radiation-related disorders (sunburn, drug phototoxic response, actinic keratosis)	1 (0.68%)
Keloid	1 (0.68%)
Connective tissue disease	1 (0.68%)
Panniculitis	1 (0.68%)
Leg ulcer	1 (0.68%)

Of the 11 patients requiring admission, the dermatological diagnoses made during teledermatology consult were re-affirmed in 10 patients in the inpatient setting and none required revision; one patient declined admission and was thereafter lost to follow-up. The two most frequent indications for admission were infective dermatological conditions requiring antibiotic administration (N = 3 (27.3%)) and complications of psoriasis (N = 3 (27.3%)). The list of the teledermatology diagnoses requiring admission is shown in Table 3. Of these patients requiring admission, 6 had known existing dermatological conditions which were consistent with their teledermatology diagnosis (psoriasis (3), lichen planus (1), eczema (2)). Two of these patients were on prior follow-up with our Dermatology unit, while 2 had their underlying dermatological conditions diagnosed elsewhere. The remaining patients presented with their dermatological condition for the first time.

**Table 3:** Teledermatology diagnoses requiring admission

Patient	Gender	Diagnosis
1	Female	Ulcerated foreign body granulomas with secondary bacterial infection
2	Male	Generalized exfoliative dermatitis with background of poorly controlled eczema †
3	Male	Erythrodermic psoriasis †
4	Female	Acute urticaria
5	Female	Contact dermatitis with secondary impetiginization and right lower limb cellulitis
6	Female	Impetiginized lichen planus †
7	Female	Flare of pustular psoriasis †
8	Female	Cutaneous small vessel vasculitis
9	Female	Flare of discoid eczema †
10	Female	Acute generalized exanthematous pustulosis
11	Female	Flare of psoriasis (absconded and did not return for in-patient review) †

† Known existing dermatological conditions which were consistent with the teledermatology diagnosis

Out of 136 patients discharged directly from ED, 129 (94.9%) patients were provided with an outpatient appointment in the dermatology clinic, out of which 10 patients (7.8%) did not come for the scheduled follow-up and 9 were instead admitted for non-dermatological reasons. Of the remaining 110 patients who were physically reviewed in-person at outpatient follow-up, 90 (81.8%) patients retained the initial teledermatology diagnoses (complete diagnostic agreement). 20 (18.2%) patients had their teledermatology diagnoses revised after in-person review, out of which 7 (6.4%) patients had their teledermatology differential diagnosis become the main diagnosis (partial diagnostic agreement) and 13 (11.8%) patients had their diagnoses completely revised (Table 4). None of the patients required admission from clinic during the outpatient follow-up appointment. All 9 of the patients who were admitted for non-dermatological reasons retained their initial teledermatology diagnosis upon in-person review in the ward. Overall complete diagnostic agreement of all patients seen via teledermatology referral was 84.5%.

## Discussion

Dermatologists review patients mainly in the outpatient clinic [2–4] and majority of the patients are managed in an outpatient setting. However, some patients may instead present to the ED for acute dermatological conditions. When an ED physician is faced with a patient with an uncommon dermatological condition or is uncertain of the management of a flare of a pre-existing skin condition, one of the disposition options is to admit the patient for definitive dermatological care. Such patients would undergo lengthy admission and clerking processes in the ward, in addition to potentially unnecessary blood tests or other investigations. Depending on the hospital structure and clinical demands of medical teams, patients may wait for up to a day before being seen by a dermatologist during an inpatient consultation round, resulting in a minimum length of inpatient hospital stay of two days. This reduces bed capacity and consumes manpower resources that could have otherwise been better utilized [4].

Teledermatology has been established as a cost-effective platform, regardless of populations engaged in its use [7]. Teledermatology reduces the number of unnecessary in-person dermatology consultations by providing specialist dermatological advice at an earlier juncture, allowing appropriate diagnostic, management and treatment advice to be administered more rapidly. The overwhelming majority of our patients (92.5%) reviewed by teledermatology could be discharged directly from ED, with only 11 (7.5%) patients being recommended for admission directly from the ED for their dermatological condition. This allows resources to be utilised for other patients more urgently requiring hospital admission for definitive care. Additionally, earlier provision of specialist dermatological review may prevent patients with severe dermatological conditions from being undiagnosed or given unsuitable follow-up appointment from the ED.

We compared the average LOS for our patients who received teledermatological consults with the LOS of patients with dermatological conditions planned to be admitted to the ward. The average LOS is 3 hours and 46 minutes for our patients for the former group, compared to 8 hours and 55 minutes for the latter group, with the longest LOS being 20 hours and 25 minutes. Several reviews found that reduction of patient’s perceived waiting time for consultations or investigations was associated with improved patient satisfaction [8, 9]. Reduction of the overall ED journey time through provision of expedited dermatology specialist input via teledermatology thus likely will contribute to better patient satisfaction by decreasing patient’s LOS.

Disregarding the point during patient’s ED stay at which the teledermatology referral was made, i.e. whether the referral was done at the outset compared to few hours after patient’s presentation to the ED, the teledermatology team was able to start reviewing the patient on average within 2 hours and 13 minutes from the start of the patient’s ED stay. Considering the hectic nature of ED with multiple inputs required from the different specialties for a given patient, this time span highlights efficiency of the teledermatology service. Crowding occurs when demands placed on the ED are greater than the hospital’s capacity to ensure timely care in the ED. Decreasing the amount of time patients spend in the ED improves patient flow within the ED [9], and avoids departmental crowding. Resources of the ED can then be utilised for other patients who require immediate medical attention and improve overall patient flow in ED.

We observed the spread of ED attendances during and outside office hours over a representative 6-month period; we found that 51% visited during office hours (9AM to 5 PM) and 49% visited outside office hours (5PM to 9AM). Extrapolating this, about half of patients attending to ED with dermatological conditions could benefit from available teledermatology service.

A study in the primary care setting by Lowell et al. [10] reported that a dermatology referral was required in 43% of patients whose skin condition was his/her chief complaint. This underscores the complementary advantage that the provision of on-demand teledermatology provides to the ED physician. A survey administered by Cheeley et al. to ED and inpatient physicians revealed that while in-patient review is preferred, the consultative teledermatology would be accepted by most providers and could be developed as a useful modality for dermatology consultation when in-patient review is not available [6]. Teledermatology provides immediate input on the dermatological condition to the ED physician; with time and repeated usage of this mode of doctor-doctor consultation, there is certain educational benefit and support to the referring ED physician [6].

For patients seen by teledermatology service, 81 (59.6%) were given outpatient follow-up within a week, while 39 (28.7%) were given appointments between 8 days and 8 weeks. Depending on the indication, dermatologists will assign appropriate duration follow-up appointment for the patients. Dermatologists thus to have better control and can optimize the number of patients seen in the outpatient clinic. This approach helps to create a more conducive environment that enables patients to receive timely and high-quality care, while also ensuring that the clinic is not overwhelmed. This approach not only benefits individual patients but also contributes to the sustainability and resilience of the broader healthcare ecosystem.

None of the patients required admission after in-person review at their outpatient follow-up appointment, in which 90 (81.8%) patients had complete diagnostic agreement and 20 (18.2%) patients with partial diagnostic agreement. This not only points to the utility of teledermatology to effectively triage patients with dermatological conditions with appropriate indication of the disposition and acuity of care required, but also reinforces of the high concordance of diagnoses. Seven patients were identified to not require any follow-up in our department, avoiding outpatient follow-up appointments altogether which would have otherwise been scheduled. The improved utilization of precious outpatient appointments in a busy outpatient dermatology clinic is yet another benefit of teledermatology. The high diagnostic agreement rates between teledermatology and in-person review demonstrated good performance and can serve to increase confidence of both the referring ED physician and the dermatologist receiving the referral.

Evidence suggests that asynchronous teledermatology results in comparable diagnostic accuracy when compared to in-person clinical consults [11]. Various studies have found the inter-observer agreement between clinic-based examiners and teledermatologists to be comparable [12, 13]. A review by Levin et al. [13] compared multiple store-and-forward studies. The diagnostic partial agreement, when including the teledermatology differential diagnoses, ranged from 50 to 100%, with the wide range potentially due to heterogeneity of cases and studies included in the review. With 84.5% overall complete diagnostic agreement between teledermatology and in-person review diagnosis, our findings likewise support this. Furthermore, all admitted teledermatology patients had their initial diagnoses affirmed in the inpatient setting after in-person review in the wards, indicating that teledermatology is sufficiently reliable in providing accurate diagnosis even in extensive and severe dermatoses with high acuity. None of the 110 patients reviewed in-person in clinic required admission after the in-patient review post ED discharge, indicating that prescribed management post-teledermatology allowed for adequate interim patient care prior to in-person review. Information about the patient’s known underlying dermatological or concomitant condition increases the accuracy of the teledermatology consult. 6 (54.5%) out of 11 patients recommended for admission after teledermatology consult had known existing dermatological conditions, knowledge of which allowed for accurate diagnosis of a flare of these conditions. Of the 110 patients who were physically reviewed in-person at outpatient follow-up, 13 (11.8%) had their teledermatology diagnoses completely revised (Table 4), with 5 patients requiring biopsies to confirm the diagnosis. In contrast, none of the patients requiring admission had their dermatological diagnoses revised. In-spite of the acceptable diagnostic accuracy of asynchronous teledermatology, there remain situations where accurate diagnosis cannot be given for dermatological diseases. This could occur when characteristic clinical features and serious complications have not yet developed or were not captured during the review. For example, a patient with a severe cutaneous adverse drug reaction could present as an exanthem to the ED but only later develop characteristic signs and symptoms of Stevens Johnsons Syndrome.

Approximately 15% of the initial teledermatology diagnoses made in ED needed to be revised after the in-person review. In-person review post asynchronous teledermatology consults in ED hence acts as a safety net in cases of diagnostic uncertainty. Increasing adequacy at the point of referral can additionally augment the diagnostic accuracy of the teledermatology consult. Development of a guide for the ED physicians focusing on the salient details required for teledermatology referrals including relevant clinical history and standardized photographic parameters would aid in this [14]. Besides increasing diagnostic accuracy, such a guide would also increase the efficiency of the teledermatology consult as it would obviate the need for repeated to and for requests for more information between the ED and Dermatology.

The main limitation of this study is its observational and descriptive nature based on teledermatology referrals from the ED. Information was retrospectively collated based on available medical records. We were unable to ascertain the total number of patients presenting to the ED with dermatological conditions in the same period. Furthermore, some patients with dermatological conditions may not have been referred to teledermatology if they present outside of the allocated service hours. We were unable to assess severity of conditions of the patients that were not referred for teledermatology and review the conditions and management of patients that were managed by the ED physicians themselves.

Teledermatology was made readily available to the ED physicians throughout office hours, and 147 patients were referred to this service over the observed period of 54 months. As the number of referrals remained in a steady state low number, the dermatology team was able to support the service without much additional clinical burden. On the other hand, the low uptake of the service translated to minimal impact to the overall admissions for patients with dermatological conditions and demand on dermatology outpatient services. Given the utility of teledermatology in ED, we will continue to encourage the uptake of teledermatology in ED in our hospital.

In conclusion, teledermatology in the setting of the ED of a tertiary hospital allows more efficient triaging of patients with dermatological conditions by providing early evaluation by a dermatologist. Patients with dermatological conditions who previously would have been admitted for inpatient care are now largely managed in the outpatient setting with appropriate specialty-directed treatment, return advice, and appropriately-triaged follow-up appointments. Measures to improve ease of use, diagnostic accuracy and reliability will encourage more routine use, availing teledermatology as a useful adjunct in practice of the dermatology in the acute and inpatient setting.

**Table 4:** Teledermatology diagnoses with revised final diagnosis after in-person review

Patient	Initial teledermatology diagnosis	Revised in-person diagnosis
1	Eczema – differential diagnosis psoriasis	Guttate psoriasis
2	Acute urticaria with flagellate component – differential diagnosis acute contact dermatitis	Contact dermatitis
3	Atypical erythema multiforme - differential diagnosis urticarial vasculitis	Urticarial vasculitis
4	Atypical erythema multiforme – differential diagnosis contact dermatitis	Contact dermatitis
5	Cellulitis – differential diagnosis cutaneous vasculitis	Cutaneous vasculitis
6	Pompholyx (not previously known)	Bullous pemphigoid (biopsy proven) †
7	Flare of atopic eczema (not previously known)	Acute contact dermatitis †
8	Eczema flare (not previously known), herpes labialis	Bullous pemphigoid (biopsy proven), chronic eczema †
9	Parainfectious exanthem	Pigmented purpuric dermatosis or cutaneous vasculitis †
10	Endogenous eczema (previously known eczema)	Inflammatory phase of BP (biopsy proven) †
11	Macerated heat rash	Staphylococcal Scalded Skin Syndrome †
12	Viral wart or eccrine poroma Hyperkeratotic lesions on the palms likely pitted keratolysis	Eccrine poroma, congenital punctate keratoderma †
13	Urticarial exanthem	Urticarial vasculitis †
14	Bullous pemphigoid	Pediculosis pubis and scabies †
15	Cutaneous small vessel vasculitis	Parainfectious erythema multiforme †
16	Pityrosporum folliculitis	Papular dermatitis - likely irritant dermatitis †
17	Acute urticaria and pompholyx – differential diagnosis bullous pemphigoid	Bullous pemphigoid (biopsy proved)
18	Pyogenic granuloma – differential diagnosis cutaneous metastasis, SCC	Melanoma (biopsy proven) †
19	Papulovesicular rash –﻿ differential diagnosis HFMD, VZV	HFMD
20	Papulovesicular eruption – differential diagnosis varicella	Scabies †

† Teledermatology diagnoses completely revised upon in-person review

## Data Availability

The datasets used and/or analysed during the current study are available from the corresponding author on reasonable request.

## List of abbreviation

ED: Emergency Department
LOS: Length-of-stay
